# Estimating Severe Coccidioidomycosis in California

**DOI:** 10.3201/eid1307.061480

**Published:** 2007-07

**Authors:** Valerie J. Flaherman, Richard Hector, George W. Rutherford

**Affiliations:** * University of California, San Francisco, California, USA; † California State University, Bakersfield, California, USA

**Keywords:** Coccidioides, coccidioidomycosis, epidemiology, severe, complications, death, dispatch

## Abstract

We used hospital discharge data to estimate incidence and distribution of coccidioidomycosis-associated hospitalizations in California. For 1997–2002, the average annual rate of hospitalization was 3.67 per 100,000 population. County of residence, older age, black race, male sex, HIV infection, and pregnancy were strongly associated with increased risk for hospitalization.

Coccidioidomycosis is caused by the fungi *Coccidioides immitis* and *C. posadasii,* which are present in soil in disease-endemic areas. Data are sparse on frequency and incidence of severe coccidioidomycosis that requires hospitalization. In California, population increase, changing racial composition, and increasing numbers of immunocompromised persons may have affected the incidence and severity of disease in recent years. We used hospital discharge data to estimate frequency and incidence of hospitalization with coccidioidomycosis for California, including its counties and its demographic subgroups.

## The Study

We examined the Inpatient Hospital Discharge Data Set from the California Office of Statewide Health Planning and Development for 1997–2002 (*1*), which contains inpatient discharge diagnoses from all nonfederal hospitals in California. We abstracted all records with any International Classification of Diseases, 9th edition (ICD-9), code for coccidioidomycosis (114–114.5 and 114.9, with 114.2 representing coccidioidal meningitis) and defined each record as a discrete hospitalization.

From each record, we extracted year of admission, county of residence, age, race, ethnicity, sex, presence of HIV infection (ICD-9 codes 042 or V08), pregnancy status (ICD9 codes V22-V23.9 or 630–676.9), vital status at discharge, and record locator number (RLN) (available for 1997–2000 only). We obtained population estimates from California Department of Finance by county of residence, age, racial-ethnic group, and sex (*2*,*3*). Numbers of AIDS cases and estimates of the population with HIV were obtained from the California Department of Health Services (*4*).

All statistical analysis was conducted by using Stata 8.2 (Stata Corp., College Station, TX, USA). We calculated the frequency of hospitalization by county, age group, racial-ethnic group, sex, pregnancy status, and immune status. Incidence of initial hospitalization for severe coccidioidomycosis was estimated by using each earliest hospitalization with a given RLN during the years 1997–2000 for which RLN was available, and rates of repeat hospitalization were calculated on the basis of subsequent hospitalizations with the same RLN. Mortality rate was calculated as crude incidence rate for death among those hospitalized for coccidioidomycosis with unique RLNs.

Bivariate relative risks (RRs) were calculated for the effect of pregnancy status and immune status on the frequency of hospitalization. Multivariate Poisson regression was used to estimate RR of year, county, age, racial-ethnic category, and sex on the frequency of hospitalization for coccidioidomcyosis. Among those hospitalized, multivariate logistic regression was used to evaluate odds ratios (ORs) for race, ethnicity, and sex on rehospitalization and meningitis.

Records for which data were missing for age, sex, race/ethnicity, or county were not included in multivariate analysis. Records that lacked data for county were not included in crude incidence calculations by county. For crude incidence calculations by age, race/ethnicity, and sex, cases for which category was unknown were redistributed among known categories in the same proportion as they occurred among those with known category. This study was approved by the Committee on Human Research, University of California, San Francisco.

From 1997 through 2002, of 7,457 inpatient hospitalizations associated with coccidioidomycosis in nonfederal institutions in California, 3,707 (50%) had a principal diagnosis of coccidioidomycosis, 1,605 (22%) had a first additional diagnosis of coccidioidomycosis, and 896 (12%) had a second additional diagnosis of coccidioidomycosis. Frequency of hospitalization for coccidioidomycosis was 3.7 per 100,000 residents per year (Table 1). Kern, Los Angeles, and San Diego counties had highest total number of hospitalizations and together accounted for 47% of all hospitalizations. There were 417 deaths, resulting in a mortality rate of 2.1 per 1 million California residents annually.

**Table 1:** Hospitalizations for coccidioidomycosis, California, 1997–2002

Category	Total hospitalizations	Total person-years (x 10^6^)	Frequency of hospitalization*	Frequency of hospitalization for coccidioidal meningitis*
Total	7,457	203.0	3.67	0.657
Year				
1997	1,269	32.5	3.90	0.706
1998	1,144	32.9	3.50	0.706
1999	1,167	33.4	3.5	0.61
2000	1,100	34.0	3.23	0.62
2001	1,291	34.7	3.7	0.58
2002	1,486	35.3	4.2	0.71
Highest incidence counties				
Kern	1,700	3.97	42.8	
Tulare	479	2.21	21.7	
Kings	133	0.77	17.4	
San Luis Obispo	170	1.48	11.5	

*Per 100,000 residents per year.

For years for which an RLN was available (1997–2000), 63% of hospitalizations were initial and 37% were repeat. The incidence of initial hospitalization for severe coccidioidomycosis was 2.4 per 100,000 residents, and 8.9% of persons initially hospitalized with coccidioidomycosis died in the initial or a subsequent hospitalization.

Pregnant women were more likely than nonpregnant women to be hospitalized with a code for coccidioidomycosis (RR 2.5, 95% confidence interval [CI] 2.03–3.08). Compared with all Californians, RR for hospitalization for persons with AIDS was 34.5 (CI 31.0–38.4) and for persons with HIV was 13.9 (CI 12.5–15.5). When only records with RLNs were examined, 24% of persons admitted with coccidioidomycosis who had HIV coinfection died during hospitalization, compared with 8.2% of persons admitted with coccidioidomycosis who did not have HIV coinfection (p<0.005 by χ^2^ analysis).

In multivariate Poisson regression that used California Department of Finance population estimates, older age, black race/ethnicity, and male sex were associated with increased risk for hospitalization. Native American and Hispanic race/ethnicity was protective for this outcome (see reference groups in Table 2). Asian-Pacific Islander race/ethnicity was protective on a statewide level but was a risk factor in the 4 counties with the highest incidence.

**Table 2:** Frequency and relative risk for hospitalization for coccidioidomycosis in California and its 4 highest-incidence counties, 1997–2002*

Population	Frequency of hospitalization†	RR for discharge diagnosis of coccidioidomycosis	Frequency of hospitalization in highest incidence counties†	RR for discharge diagnosis of coccidioidomycosis in highest incidence counties
Race‡				
White	3.6	Ref	26.6	Ref
Hispanic	3.4	0.73 (0.68–0.78)	24.1	0.71 (0.63–0.79)
Black	8	2.68 (2.48–2.91)	80.8	2.43 (2.10–2.82)
Native American	1.4	0.32 (0.21–0.51)	12.7	0.37 (0.20–0.70)
Asian-Pacific Islander	2	0.78 (0.70–0.87)	51	1.62 (1.34–1.97)
Sex‡				
Female	2.3	Ref	36.6	Ref
Male	5	2.14 (2.03–2.27)	21.9	1.67 (1.53–1.85)
Age, y‡				
<14	0.5	0.12 (0.10–0.14)	3.9	0.12 (0.09–0.15)
15–49	3.5	Ref	31.3	Ref
50–69	7.1	2.13 (2.01–2.26)	57.2	1.83 (1.66–2.03)
>70	7.3	2.74 (2.54–2.97)	47.0	1.83 (1.58, 2.12)
Special conditions§				
Pregnancy¶	3.8	2.5 (2.03–3.08)	51.9	
AIDS	127	34.5 (31.0–38.4)	912	31.0 (23.8–40.4)
All HIV	51	13.9 (12.5–15.5)	319	10.8 (8.3–14.1)

*Multivariate results reported for 6,465 cases with no missing data (87%). RR, relative risk; Ref, referent. Values in parentheses are 95% confidence intervals. †Crude incidence per 100,000 residents in Kern, Tulare, Kings, and San Luis Opispo counties. ‡RR by multivariate Poisson model controlling for year, county, age, race, and sex. For the years 1997–1999, racial-ethnic categories were White, Hispanic, Black, Native American, and Asian-Pacific Islander. For the years 2000–2002, racial-ethnic categories were White, Hispanic, Black, Native American, Asian, Pacific Islander, and multirace. The population of multirace represented ≈1% of populations relevant to the study and was not included in the analysis. California Department of Finance population estimates from 2000–2002 for Asians and Pacific Islanders were combined into a single category, Asian-Pacific Islander, to match coding of the Office of Statewide Health Planning and Development database. All Hispanics in the Department of Finance data were assumed to be of white race. §Bivariate relative risk. ¶To estimate a denominator for the pregnant population, we estimated the total person-years of pregnancy for each county in California in the following manner. The total number of live births was multiplied by 0.75 (to approximate 9 mo of pregnancy) and added to the total number of fetal deaths, multiplied by 0.56 (to estimate a gestation of 30 weeks). This sum was finally added to the total number of abortions, multiplied by 0.19 (to estimate a gestation of 10 weeks). Estimates of live births and fetal deaths were obtained from the Center for Health Statistics Birth Rate Tables (*5*). Annual number of abortions was estimated from federal abortion surveillance data from 1997 (*6*).

Logistic regression showed that black persons hospitalized with a diagnosis of coccidioidomycosis had increased risk for rehospitalization (OR 2.08, CI 1.59–2.73) compared with white persons, controlling for year, county, age, and sex. Controlling for the same confounders, Asian-Pacific Islanders hospitalized with coccidioidomycosis had increased risk for meningitis (OR 1.63, CI 1.02–2.63); Hispanic race/ethnicity was protective against meningitis (OR 0.63, CI 0.48–0.84).

## Conclusions

Hospitalizations for coccidioidomycosis are common in California, especially in disease-endemic areas. Deaths from coccidioidomycosis average ≈70 per year statewide. Persons with AIDS have both a very high frequency of hospitalization for coccidioidomycosis and a very high proportion of deaths from the disease. Persons with AIDS in the 4 counties with the highest frequency of coccidioidomycosis (Kern, Tulare, Kings, and San Luis Obispo) have a frequency of hospitalization for coccidioidomycosis that approaches 1% per year. This study confirms several well-known risk factors for coccidioidomycosis, including black race, middle age and older age, and pregnancy (*7*). We did not find evidence supporting previous reports of Hispanic and Asian racial/ethnic background as a risk factor for coccidioidomycosis hospitalization statewide (*8*,*9*).

This study has several limitations. First, we included all hospitalizations that contained any discharge diagnosis of coccidioidomycosis; 73% of our included hospitalizations coded coccidioidomycosis as principal or first additional diagnosis. Among those hospitalizations for which coccidioidomycosis was not principal or first additional diagnosis, it is unclear whether other principal diagnoses (such as AIDS) would have caused hospitalization in the absence of coccidioidomycosis. In this respect, our findings may overestimate the incidence of disease. Second, we have no information about persons hospitalized in federal hospitals, which could lead to an underestimation of effects of disease or bias of results regarding disease distribution. However, because only 19 of 570 hospitals licensed in California in 1999 were federal, the bias of omitting federal hospitals is likely to be small (*10*). Third, we had information on duplicate hospitalizations for 1997–2000 only, and some persons either may have had a repeat hospitalization subsequently or may have been hospitalized for coccidioidomycosis before this period. Fourth, we have no data on the subcategory of persons with Asian ancestry. The increased risk we found for Asian-Pacific Islanders in the 4 counties with highest incidence is not consistent with risk statewide, and this might be partly explained by differential risk by subcategory of race/ethnicity. However, differential exposure patterns based on employment or recreation might also contribute to the discrepancy. Further research in this area is needed.

The risk for severe disease and death attributable to coccidioidomycosis in California is of a magnitude similar to the risk from varicella in the state before the varicella vaccine (*11*,*12*). Furthermore, a substantially higher risk exists for many subgroups, including residents of high-incidence counties, middle-aged and older persons, pregnant women, black residents, and those with HIV infection. Recent progress in vaccine development has raised the possibility of a way to better control this disease (*13*). The development of new therapeutic and preventive modalities could do much for the population at risk and should be considered a priority for healthcare research. Our data offer a good overall estimate of the incidence of severe disease for the state, but to assess the success of a new vaccine, further studies will need to determine initial hospitalization and primary cause of hospitalization with greater specificity.
